# An Efficient Feature Selection Strategy Based on Multiple Support Vector Machine Technology with Gene Expression Data

**DOI:** 10.1155/2018/7538204

**Published:** 2018-08-30

**Authors:** Ying Zhang, Qingchun Deng, Wenbin Liang, Xianchun Zou

**Affiliations:** ^1^College of Computer and Information Science, Southwest University, Chongqing 400715, China; ^2^Department of Gynecology and Obstetrics (Southwest Hospital), Third Military Medical University, Chongqing 400038, China; ^3^Key Laboratory of Luminescent and Real-Time Analytical Chemistry (Southwest University), Ministry of Education, College of Chemistry and Chemical Engineering, Southwest University, Chongqing 400715, China

## Abstract

The application of gene expression data to the diagnosis and classification of cancer has become a hot issue in the field of cancer classification. Gene expression data usually contains a large number of tumor-free data and has the characteristics of high dimensions. In order to select determinant genes related to breast cancer from the initial gene expression data, we propose a new feature selection method, namely, support vector machine based on recursive feature elimination and parameter optimization (SVM-RFE-PO). The grid search (GS) algorithm, the particle swarm optimization (PSO) algorithm, and the genetic algorithm (GA) are applied to search the optimal parameters in the feature selection process. Herein, the new feature selection method contains three kinds of algorithms: support vector machine based on recursive feature elimination and grid search (SVM-RFE-GS), support vector machine based on recursive feature elimination and particle swarm optimization (SVM-RFE-PSO), and support vector machine based on recursive feature elimination and genetic algorithm (SVM-RFE-GA). Then the selected optimal feature subsets are used to train the SVM classifier for cancer classification. We also use random forest feature selection (RFFS), random forest feature selection and grid search (RFFS-GS), and minimal redundancy maximal relevance (mRMR) algorithm as feature selection methods to compare the effects of the SVM-RFE-PO algorithm. The results showed that the feature subset obtained by feature selection using SVM-RFE-PSO algorithm results has a better prediction performance of Area Under Curve (AUC) in the testing data set. This algorithm not only is time-saving, but also is capable of extracting more representative and useful genes.

## 1. Introduction

Cancer has become the main cause of morbidity and mortality worldwide, due to population growth, aging, and the spread of risk factors such as tobacco, obesity, and infection, which will worsen in the next decade, in which breast cancer is the most common cancer, especially in women [1–8]. At present, the treatment of breast cancer is seriously lagging behind. Although a number of effective measures have been identified, it is hoped to identify the tumor and prepare for further diagnosis [9–14]. However, as a late intervention method, the effect is still limited. The generation and development of tumor are closely related to genes, and, from gene level to cancer diagnosis, can also be detected by the gene [14–18]. For example, Karlsson A et al. [19] applied unsupervised analysis of gene expression data and identified a phenotype comprising 90% of 2015 world health organization (WHO) lung cancer. Molina-Romero C et al. [20] pointed out that the current classification of lung cancer has greatly changed the pathological diagnosis of infiltrating adenocarcinoma of the lung and identified a subtype of disease which has a significant impact on medical practice. But for the time being, there are still obvious deficiencies in the study of breast cancer. As a method of early prediction and risk assessment, machine learning can reduce the incidence of cancer in a simpler and more effective way, thereby reducing the pain of patients and improving the quality of human life. The prediction of breast cancer genes is still critical, which can improve breast cancer prediction, interfere with the treatment as soon as possible, and reduce the incidence of breast cancer, thereby further improving the quality of human life.

A genetic disease may produce or cause one or more mutations; this phenomenon also reflects the characteristics of the real biological system module [21]. Analysis of individual genes often does not reflect gene interactions, nor does it make it possible for multiple genotypes to predict disease. Over the last decade, genome-wide array based expression profiling studies have been used as a forceful tool to improve biological understanding of breast cancer. With these techniques, many prognostic signatures of gene expression have been identified as predictive of breast cancer recurrence risk [22]. Dr. Golub [23] and his research team successfully selected 50 gene subsets from 7129 genes of leukemia gene expression data by means of signal-noise ratio, which enabled the accurate classification of experimental samples. Feature extraction methods for cancer classification using gene expression data include principal component analysis (PCA) [24], independent components analysis (ICA) [25], linear discriminant analysis (LDA) [26], local linear embedding (LLE) [27], and partial least squares regression (PLS) [28]. Huang and Zheng [29] combined PCA and LDA algorithms and proposed a new feature extraction method. The method of decision is principal component analysis. The method of decision is principal component analysis. Support vector machine recursive feature elimination (SVM-RFE) approach was originally proposed by Guyon [30], which can effectively extract the informative genes for cancer classification. SVM-RFE is used to find the discriminating relationship within the clinical data sets and within the gene expression data sets formed by tumor arrays and normal tissues. Liu ShenLing [31] proposed that the algorithm considers that the use of RFE algorithm can ensure the fact that the feature subset is preserved during feature ordering. This method uses the information of the discriminant function of the SVM to rank the features.

In this paper, based on traditional gene selection algorithms, we proposed a model combined with grid search method and heuristic algorithms, namely, genetic algorithm and particle swarm algorithm, to search for the best parameters for the gene selection algorithms. The important genes within breast cancer gene expression data extracted from SVM-RFE-GS, SVM-RFE-PSO, and SVM-RFE-GA algorithms were regarded as the final features, and samples with these features were fed into classifier to train it. By comparing the classification accuracy of the above three algorithms, it could be concluded that the SVM-RFE-PSO algorithm could be better applied to select genes from breast cancer gene expression data than traditional SVM-RFE algorithm, and the SVM-RFE-PSO algorithm can select fewer gene features but can extract more information effectively. We also used RFFS, RFFS-GS, and mRMR algorithm for comparative experiments in feature selection and found that the SVM-RFE-PSO algorithm is still better than other algorithms. In addition, the classification accuracy using SVM-RFE-PSO algorithm is the best one. This method can also be used for more feature selection problems.

## 2. The Proposed Method

### 2.1. Feature Selection

#### 2.1.1. Statistics

Cancer gene expression data are highly dimensionally characterized, so it is important to filter out differential genes in the sample data.* p* value can usually be calculated for each gene by statistical methods such as T-test, which is commonly used in differential gene expression testing. It evaluates whether a gene is differentially expressed in two samples by combining variable data between samples. However, due to the limited amount of experimental samples, the estimation of overall variance is not rather accurate. So the test performance of statistical test is reduced. Furthermore, the false positive rate (FPR) would increase significantly if the statistical test is repeated. In order to control the number of the FPR, we need to test the* p* value for multiple tests to increase the threshold.

The false discovery rate (FDR) error control method was proposed by Benjamin and Hochberg in 1995 [32] to determine the range of* p* values by controlling the FDR value. The steps to filter out the differential genes are as follows. First, we calculate* p* value for each gene. Then we calculate the value of FDR. Finally, we use the FDR error control method to make multiple hypothesis tests for* p* value. The adjusted* p* value,* q*-value, is obtained by FDR control. The smaller the* q*-value, the more obvious the difference between the genes is, so we should try to choose the smaller* q*-value gene as the differential gene.

#### 2.1.2. SVM-RFE Based Model

The SVM-RFE algorithm constructs a ranking coefficient according to the weight vector *ω* generated by the SVM during training, removes a signature attribute with the smallest ranking coefficient in each iteration, and finally obtains the decreasing order of all signature attributes. Fan Zhang et al. [33] used the SVM-RFE algorithm to remove the features of the gene expression data to form a new feature set.

Enter the training samples {*x*_*i*_, *y*_*i*_}, *yi* ∈ {−1, +1}. The output feature ordering set is defined as *R*.

(1) Initialization. The original feature set *S* = 1,2,…, *D*; the feature ordered set *R*= *∅*.

(2) Loop the following procedure until *R*= *∅*.

(a) Obtain the training set with candidate feature set.

(b) Train the SVM classifier to get *ω*.

(c) Calculate the ranking criteria score:

(1) $$\begin{array}{c} c_{k}=\omega_{k}^{2},\;k=1,2,\ldots ,\left|S\right| \end{array}$$

(d) Find the smallest ranking criteria score features:

(2) $$\begin{array}{c} \underset{k}{\arg\min}\;c_{k}. \end{array}$$

(e) Update feature set *R* = *P* ∪ *R*.

(f) Remove this feature in *S* such that *S* = *S*/*P*.

#### 2.1.3. RFFS Based Model

The random forest (RF) is an ensemble learning algorithm which constructs many decision trees at training time and according to the results of its trees outputs the final classification results. When applied to classification tasks, one of the important attributes of RF was that it could compute the significance of the attributes [34].

RFFS is a wrapper feature selection method based on random forest. It uses the variable importance of random forest algorithm to sort the features and then uses the sequence backward search method. Each time the feature set is removed, the least important it is (the least importance score is the smallest). The characteristics are successively iterated, and the classification accuracy rate is calculated. Finally, the feature set with the least number of variables and the highest classification accuracy is obtained as the feature selection.

Input: The original feature set.

Output: The maximum classification correct rate on the verification data set *TGMax* and its corresponding feature set *FGSort*.

Step:

*(1) Initialization*

(1) Read in the original data set S.

(2) Set *TGMax* = 0.

*(2) For (ft in N-2)*

(1) Randomly divide the data set S into 10 equal parts.

(2) Set local maximum classification accuracy *TLMax* = 0.

(3) Set the local average classification accuracy rate *TLMean* = 0.

(4) Initialize the classification accuracy of each iteration in 10-fold cross-validation *Acc*[1 : 10] = 0.

(5) For(i in 1:10)

Create a classifier by running random forest on S.

Perform predictor on the test data set for classification.

Compare classification results with observations and calculate *Acc*.

Compute *TLMean*=*TLMean* + *Acc*[*i*]/10.

If(*TLMax* < = *Acc*[*i*]).

Then *TLMax* = *Acc*[*i*].

Sort features by variable importance and save as *Sort*.

(6) If(*TGMax* < = *TLMean*)

Then *TGMax* = *TLMean*,*FGSort* = *Sort*.

(7) Remove the feature with the lowest importance score from *Sort* and get the new data set *S*.

*(3) Output Result*

(1) Output global highest classification accuracy *TGMax*.

(2) Output the global maximum classification accuracy corresponding to the feature set *FGSort*.

#### 2.1.4. Minimal Redundancy Maximal Relevance Based Model

Minimum redundancy maximum redundancy (mRMR) is a filter type feature selection method, mainly by maximizing the correlation between the features and the classified variables, and minimizing the correlation between features to get the best feature set. mRMR algorithm guarantees maximum correlation while removing redundant features. In the obtained feature set, there is a great difference between the features, and the correlation with the target variables is also very large [35, 36].

Our goal is to select a set of influential features by using the mRMR algorithm. The remaining question is how to determine the best number of features. Since the mechanism of removing potential redundancy from potential features is not considered in the incremental selection, we need to refine the results of incremental selection based on the idea of mRMR. In the first stage, we use mRMR algorithm to find candidate feature sets. In the second stage, we use more complex mechanisms to search the feature subset from the candidate feature set [37].

In order to select candidate feature sets, we calculated a large number of cross-validation classification errors and found a relatively stable small error range. This range is called *Ω*. The best characteristic number of candidate set (expressed as n*∗*) is determined in *Ω*.

(1) Use mRMR incremental selection to select n (a present large number) sequential features from the input X. This leads to n sequential feature sets *S*_1_ ⊂ *S*_2_ ⋯ ⊂*S*_*n*_.

(2) Compare the feature sets of n sequence *S*_1_, ⋯, *S*_*n*_, (1 ≤ k ≤ n) to find the range of k, which is called *Ω*; the corresponding (cross-validation classification) error *e*_*k*_ is always small (i.e., with small mean and small variance).

(3) Find the smallest classification error *e∗* = min⁡*e*_*k*_ within *Ω*. The best size of the candidate feature set is *n∗*, which is selected as the smallest k and responds to e*∗*.

#### 2.1.5. Models Parameters Optimization

Grid search is similar to the exhaustion method. It is to find all the combinations and experiments. During the experiment, we need to cross-validate. Taking five-level cross-validation as an example, for each possible combination of parameters, one-fifth of the sample is used for testing, and the others are used for training. Average after five cycles, the final set of parameters with the smallest cross-validation error is the optimal parameter pair we want.

Unlike the exhaustive search, heuristic search means that the search in the state space evaluates the position of each search to get the best position and then searches from this position to the target. Some parts of information generate inferences for calculations. Particle swarm optimization algorithm (PSO) is a kind of heuristic algorithm based on swarm intelligence, which originated from the research of bird predation behavior; the basic idea of the algorithm is through collaboration and information sharing between individual groups to find the optimal solution [38]. The genetic algorithm (GA) is a reference to natural selection and evolution mechanism development of highly parallel, randomized, adaptive search algorithm. In the early days, it was an attempt to explain the complex adaptation process of the biological system in the natural system and simulate the mechanism of biological evolution to construct the model of the artificial system [39]. In this paper, the heuristic algorithm PSO and GA algorithm are mainly used for parameter optimization in order to obtain the optimal parameter optimization algorithm.

For SVM, there are many kernel functions to choose, and different kernel functions correspond to different feature maps, so the SVM hyperplane also has different abilities and characteristics. In this paper, the data used have the characteristics of small sample, high dimension, and nonlinearity, so the RBF kernel function is selected in this experiment. The RBF kernel function contains two parameters c and g, so the above algorithms, namely, GS, PSO, and GA were used to find the optimal parameter pair (c, g) in the process of building SVM classifier with cross-validation.

For random forest, the choice of random forest parameters is critical to performance. For example, a random forest allows a single decision tree to use the maximum number of features, which we called *max*-*features*. Increasing the *max*-*features* generally improves the performance of the model, but at the same time it reduces the diversity of individual trees and slows down the algorithm. The number of trees in a random forest, *n*-*estimators*, is also important for the performance of random forests. When there are many decision trees in a random forest, both the space and time complexity would be greater. This paper uses the grid search method to search for the best parameters (*max*-*features*, *n*-*estimators*) of the model, which we called RFFS-GS algorithm.

Generally, the method initializes the required set of features into the entire set of genes, and at each iteration, the feature with the smallest ordering coefficient is removed and the rest of the features are then retrained to obtain a new ordering coefficient. By iteratively executing this process, a feature ordering table can be obtained using feature selection method combined with parameter optimization models. Different parameter optimization models are used to find the best parameters for different feature selection methods. The sorted list defines a number of nested feature subsets to train feature selection models, and the prediction accuracies of these subsets were used to evaluate the advantages and disadvantages of these subsets so as to obtain the optimal subset of features.

It should be noted that each of the features listed above is used separately and may not necessarily result in the best classification performance of the SVM classifier, but the combination of multiple features will enable the classifier to obtain the best classification performance. Therefore, the proposed gene selection algorithms can choose a complementary feature combination.

### 2.2. Classification

Support vector machine is often used in two-class classification tasks, its basic model is to find the best separating hyperplane on the feature space, so that the positive and negative sample intervals on the training set are the largest.

The basic idea of support vector machine is as follows: firstly, search for the optimal hyperplane of two types of samples in the original sample space under the linearly separable samples. The classification hyperplane is established by SVM, which can ensure the classification accuracy and maximize the margin on both sides of the hyperplane including the maximization of interval, so as to realize the optimal classification of linear separable problems.

Given the set of training samples on a feature space *D* = {(*x*_1_, *y*_1_), (*x*_2_, *y*_2_),…, (*x*_*n*_, *y*_*n*_)}, *y*_*i*_ ∈ {−1, +1}, *i* = 1,2,…, *n*, *n*, represents the sample size. Thus, the objective function can be defined as

(3) $$\begin{array}{c} \max\gamma \end{array}$$

where *γ* is the geometric interval. Additionally, we also need to meet some conditions; according to the definition of interval we can see the following:

(4) $$\begin{array}{c} y_{i}\left(\;\omega^{T}x_{i}+b\;\right)=\gamma_{i}\geq \gamma ,\;i=1,\ldots ,N \end{array}$$

After a series of formulas are derived, the above objective function is transformed into

(5) $$\begin{array}{c} \max\frac{1}{\left\|\omega \right\|},\\ y_{i}\left(\;\omega^{T}x_{i}+b\;\right)\geq 1,\;i=1,\ldots ,N \end{array}$$

By solving this problem, the SVM classifier could be obtained. When a sample is linearly inseparable or does not know in advance whether it is separable, the training sample cannot satisfy the condition *y*_*i*_(*ω*^*T*^*x*_*i*_ + *b*) ≥ 1, *i* = 1,…, *N*. In this case, we can introduce slack variables, that is, to allow mis-divided samples, and the optimal hyperplane obtained at this time becomes the generalized classification hyperplane. So after we add a relaxation term to the equation, the equation could become

(6) $$\begin{array}{c} y_{i}\left(\;\omega^{T}x_{i}+b\;\right)+\varepsilon_{i}\geq 1,\;i=1,\ldots ,N \end{array}$$

Obviously when the division is wrong, the error *ε*_*i*_ is greater than zero. Introducing the error penalty coefficient C, the generalized optimal classification surface problem can be further evolved to the minimum of the following functions under the above constraints:

(7) $$\begin{array}{c} \min\left(\;\frac{1}{2}{\left\|\;\omega \;\right\|}^{2}+C\left(\;\overset{n}{\underset{i=1}{\sum }}\varepsilon_{i}\;\right)\;\right),\\ y_{i}\left(\;\omega^{T}x_{i}+b\;\right)+\varepsilon_{i}\geq 1,\;i=1,\ldots ,N \end{array}$$

The penalty coefficient C is used to control the degree of penalty for misclassified samples and to achieve a tradeoff between the proportion of misclassified samples and the complexity of the algorithm. The bigger the C, the heavier the penalty for error.

When the original problem is converted into a duality problem, the original formulas become

(8) $$\begin{array}{c} \frac{1}{2}\overset{n}{\underset{i=1}{\sum }}\;\overset{n}{\underset{j=1}{\sum }}y_{i}y_{j}\alpha_{i}\alpha_{j}\left(\;X_{i}X_{j}\;\right)\\ \overset{n}{\underset{i=1}{\sum }}\alpha_{i},\;\left(i=1,2,\ldots ,n\right)\\ \overset{n}{\underset{i=1}{\sum }}y_{i}\alpha_{i}=0,\;0\leq \alpha_{i}\leq C, \end{array}$$

If the original data is nonlinear, we map it to a high dimensional space, and the data becomes linearly separable. The kernel function does not need to explicitly map the samples in the input space into the new space and can directly calculate the inner product *ϕ*(*x*_*i*_)*ϕ*(*x*_*j*_) in the input space. It is an implicit mapping of the input space to a high dimensional space. It does not need to explicitly give that mapping, and *ϕ*(*x*_*i*_)*ϕ*(*x*_*j*_) can be calculated in the input space. In this way, the optimization problem becomes as follows:

(9) $$\begin{array}{c} \frac{1}{2}\overset{n}{\underset{i=1}{\sum }}\;\overset{n}{\underset{j=1}{\sum }}y_{i}y_{j}\alpha_{i}\alpha_{j}K\left(\;X_{i}X_{j}\;\right)-\overset{n}{\underset{i=1}{\sum }}\alpha_{i}\;\left(i=1,2,\ldots ,n\right)\\ \overset{n}{\underset{i=1}{\sum }}y_{i}\alpha_{i}=0,\;0\leq \alpha_{i}\leq C. \end{array}$$

## 3. Results and Discussion

### 3.1. Data

Gene expression data contains DNA microarray data and RNA-seq data. Analysis of microarray data helps clarify biological mechanisms and push drugs toward a more predictable future. Compared to hybridization-based microarray technology, RNA-seq has a larger range of expression levels, and more information is detected. In this paper, DNA microarray data and RNA-seq data were used for research.

The DNA microarray data used in the experiment is peripheral blood data with the accession number GSE16443 under the public database GEO platform. All of them were taken with URL https://www.ncbi.nlm.nih.gov/geo/download/?acc=GSE16443 with minor modification for employed program. The data set includes 130 sample data containing 67 breast cancer samples and 63 control samples. After we randomly divided the 130 samples into two groups, we obtained 32 health samples and 33 cancer samples in the training set and 31 health samples and 34 cancer samples in the testing set. The division of data sets can be seen from Table 1.

The RNA-seq data used in the experiment is RNA-seq data of breast cancer through the TCGA database, which were taken with URL http://portal.gdc.cancer.gov/ with minor modification. The data set includes 1178 sample data containing 1080 cancer samples and 98 health samples. We randomly divided the data set into a training set and a test set. The training set and the test set each contain 540 cancer samples and 49 health samples. The division of data sets can be seen from Table 2.

### 3.2. Experimental Results

For DNA microarray data, we calculated the FDR q-value using the package “Q-value” of R; the* q*-value distribution of GEO platform data is shown in the Table 3. From the table, we found that the differences between the genes are not particularly noticeable. In this study,* q*-value <0.1 has no genes, so* q*-value <0.2 is selected as the differential gene after screening.

For RNA-seq data, we used the 'edgeR' package of R to calculate the FDR q-value. We chose a significance screening filter q-value<0.001.

When using the SVM algorithm for classification directly, we use all the differential genes screened as feature subsets. After using the feature selection algorithm, we can get an ordered set and then we loop through the genes in the ordered set as feature subsets and select the subset with the best classification effect and the fewer features as the final feature subset. When we get the final subset of features, we use the SVM classifier for classification.

From Tables 4 and 5, we can see the classification effect obtained by using SVM algorithm and SVM-RFE algorithm on GEO data set and TCGA data set. From Table 4 we can see that using the SVM-RFE algorithm when we selected 12 genes to classify, a higher classification accuracy of 78.4615% was obtained comparing to the direct expression data of 56 genes using SVM algorithm. From Table 5, we can see that when we selected 15 genes for classification, the SVM-RFE algorithm was used to obtain a higher classification accuracy of 91.5110% compared with the direct expression data of 159 genes using the SVM algorithm. But there are still improvements for cancer classification work. Therefore, the idea of parameter optimization is introduced by improving the algorithm in the feature elimination stage, and the optimal classification parameters are found and the optimal classification model is obtained by selecting different parameter optimization methods.

**Figure 1:**
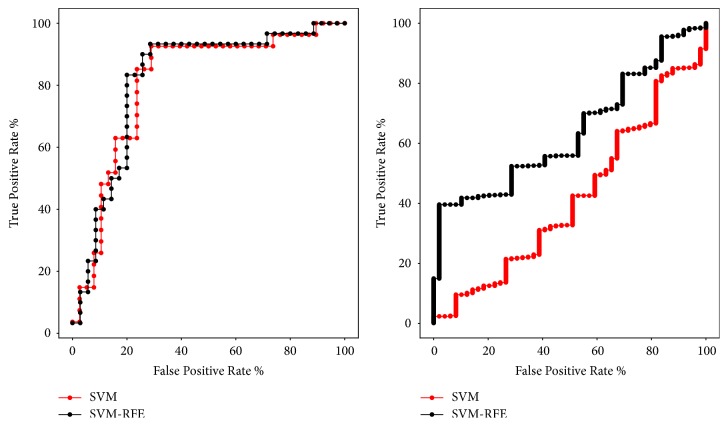
ROC curves obtained using the SVM and SVM-RFE algorithm on GEO data set (left) and TCGA data set (right).

In Figure 1, We compared ROC curves of SVM and SVM-RFE algorithm on GEO data set and TCGA data set. In other words, we compare the ROC curves of using SVM-RFE algorithm for feature selection and no feature selection. As can be seen from the figure, the area under the ROC curve (AUC) obtained after feature selection using the SVM-RFE algorithm has increased.

There are three main methods of parameter optimization, in which the grid optimization algorithm is the most common one. Similar to the exhaustive search, grid search attempts all possible (c, g) pairs of values and then cross-validates to find the (c, g) pairs with the highest cross-validation accuracy as the optimal parameter.

Although the highest classification accuracy could be obtained in the cross-validation, since using grid search included the global optimal solution, it may be time-consuming to find the best parameters c and g in a larger range. Using the heuristic algorithm, all the parameters could be traversed in the grid for the global optimal solution.

By using the heuristic algorithm PSO and GA algorithm to optimize the parameters, the optimal parameters are applied to the classification of cancer. Using these two algorithms, we can get the optimized parameters in the feature elimination stage and get the optimal classifier, so as to get the parameter w to eliminate features. Therefore, we call the feature elimination algorithm SVM-RFE-PSO and SVM-RFE-GA, respectively. We use these two algorithms to get the feature subset and classify them by SVM classifier and compare their advantages and disadvantages through classification results.

**Figure 2:**
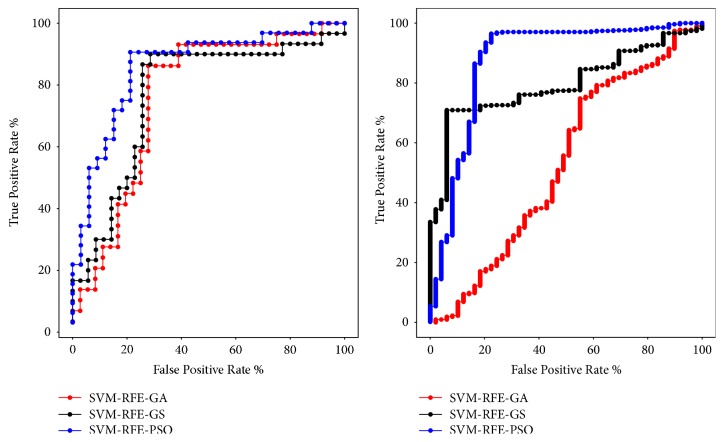
ROC curves obtained using the SVM-RFE-GS, SVM-RFE-PSO, and SVM-RFE-GA algorithm on GEO data set (left) and TCGA data set (right).

As shown in Tables 6 and 7, the classification accuracy obtained by SVM-RFE-GS algorithm is 78.4615% and 91.0017%. The classification accuracy obtained by SVM-RFE-PSO algorithm is 81.5385% and 91.6808%, and the classification accuracy of SVM-RFE-GA algorithm is 76.9231% and 91.3413%. Among them, the effect of using SVM-RFE-PSO algorithm is the best one on both data sets, and the result of using SVM-RFE-GA algorithm is the worst one in the studied algorithms on GEO data set. On TCGA data set, although the classification accuracy of SVM-RFE-GA is better than SVM-RFE-GS, the AUC area obtained by using the SVM-RFE-GS algorithm is larger. In Figure 2, we can also see that the area under the ROC curves (AUC) obtained by the SVM-RFE-PSO algorithm is the largest.

**Figure 3:**
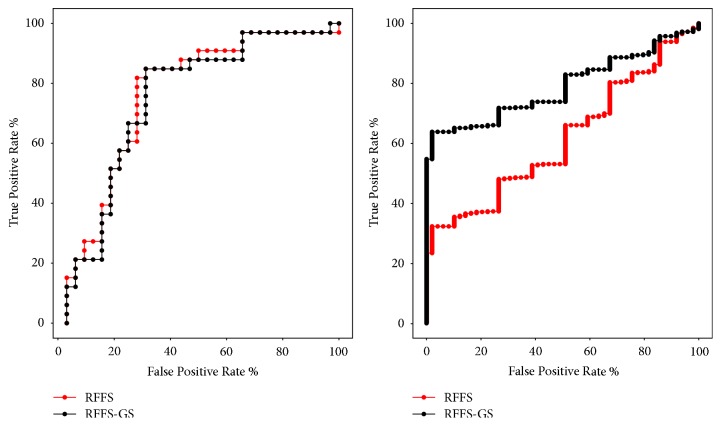
ROC curves obtained using the RFFS and RFFS-GS algorithm on GEO data set (left) and TCGA data set (right).

The RFFS and mRMR algorithms are often used for feature selection and both can get a sorted subset in the feature selection process. RFFS-GS optimizes the parameters of the RFFS algorithm by using the grid optimization method. The method of parameter optimization often achieves better results. In this paper, we use these three methods to select features of two data sets and verify their effects by classification. In Figure 3, we can see that the effect of RFFS and RFFS-GS algorithms on the GEO data set is not much different, but on the TCGA data set, the effect of the RFFS-GS algorithm is significantly better than the RFFS algorithm. From Tables 8 and 9, we can see the classification performance of the above three methods. On GEO data set, the classification accuracy obtained by using RFFS-GS is 80%, which is the best one, but the AUC area obtained by using RFFS is the best, and the classification accuracy and the AUC of mRMR algorithm are the worst. On TCGA data set, the classification accuracy obtained by using RFFS-GS is better, and the AUC of mRMR algorithm is the best one.

**Figure 4:**
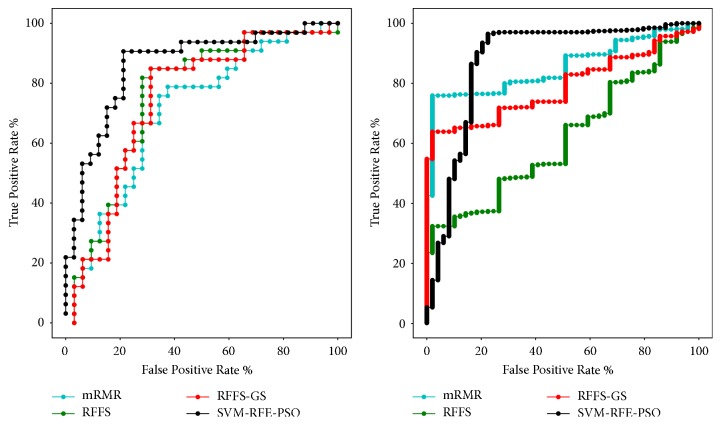
ROC curves obtained using the SVM-RFE-PSO, RFFS, RFFS-GS, and mRMR algorithm on GEO data set (left) and TCGA data set (right).

From the above, we can conclude that the SVM-RFE-PO algorithms perform better on GEO data set. And on TCGA data set, SVM-RFE-GS algorithm and SVM-RFE-PSO algorithm also have a good performance. After we compare the AUC obtained by the SVM-RFE-PSO, RFFS, RFFS-GS, and mRMR algorithms of the two data sets, in Figure 4, we can see that the AUC of SVM-RFE-PSO is the largest one. Combining the above methods, it could be found that the AUC area obtained by using the SVM-RFE-PSO algorithm is the largest, and the highest classification performance is obtained by using the algorithm for classification. Moreover, the number of genes selected in this algorithm is only eight on GEO data set and six on TCGA data set and could represent most of the information of the original set of genes.

For gene microarray data, there have been many related feature selection methods.  Table 10 lists some feature selection methods and their effects on the same data set. It can be seen that the SVM-RFE-PSO algorithm has advantages in better feature selection.

The eight gene names and their IDs are obtained by using the SVM-RFE-PSO algorithm on GEO data set which are shown in Table 11. And from Table 12, we can get the six gene names obtained by using the SVM-RFE-PSO algorithm on TCGA data set.

As can be seen from Table 13, the genes extracted using the two data sets did not overlap. This may be related to the nature of the two data sets.

The GEO data set used in this paper is the gene expression data obtained based on microarray technology, and the number of samples in this data set is small. When the number of samples is small, the increase and decrease of the sample will significantly affect the accuracy of the algorithm. The GEO data set has fewer differentially expressed genes, and the differences between genes are not obvious. There are no genes with* q*-value <0.1. When we perform differential gene filtering, errors may occur. The TCGA data set used in this paper is based on the gene expression data obtained by RNA-seq technology. The number of samples is larger, the number of differential genes is more, and the difference between genes is more obvious.

Prior to RNA-seq technology, microarray technology was the mainstream technology for studying gene expression profiles. However, when quantifying gene expression, microarray technology is limited to gene levels. RNA-seq high-throughput sequencing technology is now commonly used in biology. RNA-seq can be used for unspecified genes and subtypes of any species, allowing genome-wide analysis of any species, while microarray technology relies on prior information to quantify known genes. In addition, RNA-seq has very low background noise and high sensitivity, while having a larger detection range. The study found that the RNA-seq technology detected that the number of differentially expressed genes was about twice that of the microarray technology.

The genes expressed by cells in different environments are different, and RNA-seq can provide real-time expression of genes rather than information fixed in the genome. RNA-seq can compare changes in expression profiles of tumors in different drugs and treatments and provide more information than changes in genome exomes. Therefore, genes screened using RNA-seq data may have higher confidence, and the use of RNA-seq gene expression data will have more significance for future research on cancer.

## 4. Conclusions

We developed an integrated method in the early detection of breast cancer. In view of the high dimension of gene expression data, some methods were selected to reduce the dimension. First of all, statistical methods and FDR error control method were employed to screen genes. After FDR control, 56 genes with* q*-value less than 0.2 were selected as the characteristic genes on GEO data set and 159 genes with* q*-value less than 0.001 were selected on TCGA data set, which were used in the SVM-RFE algorithm. As a result, the effect of SVM-RFE method could be further improved. Therefore, we use the GS algorithm, the PSO algorithm, and the GA algorithm, respectively, to search the SVM kernel parameters in the feature elimination stage and obtain the feature subsets. We also use RFFS, RFFS-GS, and mRMR algorithm for comparative experiments in feature selection. It is observed from the results that SVM-RFE-PO emerges as a potentially dominant feature extraction technique for gene expression data classification. The SVM-RFE-PSO algorithm is able to extract the optimal discriminative feature information gene from the expression data. We use these characteristic information genes to study the relationship between genes and cancer. This has an extremely important role in the in-depth discovery and understanding of the disease mechanism and in the improvement of the clinical diagnostic accuracy of the disease. In the future, we will explore more feature extraction algorithms to achieve more accurate feature screening.

## Figures and Tables

**Table 1:** Division of DNA microarray data on GEO data set.

	Health	Cancer	Total
Training set	32	33	65
Testing set	31	34	65
Total	63	67	130

**Table 2:** Division of RNA-seq data on TCGA data set.

	Health	Cancer	Total
Training set	49	540	589
Testing set	49	540	589
Total	98	1080	1178

**Table 3:** Division of training and testing set on GEO data set.

	<0.01	<0.05	<0.1	<0.2	<0.3	<1
p value	406	1326	2200	3719	5065	11216
q-value	0	0	0	56	643	11216

**Table 4:** The performance comparison of SVM and SVM-RFE algorithm on GEO data set.

Measure	SVM	SVM-RFE
genes	56	12
Accuracy	76.9231%	78.4615%
Precision	67.6471%	73.5294%
Recall	85.1852%	83.3333%
F-score	75.4098%	73.5294%
AUC	0.8080	0.8181

**Table 5:** The performance comparison of SVM and SVM-RFE algorithm on TCGA data set.

Measure	SVM	SVM-RFE
genes	159	15
Accuracy	91.6808%	91.5110%
Precision	100%	99.8148%
Recall	91.6808%	91.6667%
F-score	95.6599%	95.5674%
AUC	0.41565	0.63938

**Table 6:** The performance comparison of SVM-RFE-GS, SVM-RFE-PSO, and SVM-RFE-GA algorithm on GEO data set.

Measure	SVM-RFE-GS	SVM-RFE-PSO	SVM-RFE-GA
genes	8	8	8
Accuracy	78.4615%	81.5385%	76.9231%
Precision	73.5294%	79.4118%	70.5882%
Recall	83.3333%	84.3750%	82.7586%
F-score	78.125%	81.8182%	76.1905%
AUC	0.7686	0.8589	0.7605

**Table 7:** The performance comparison of SVM-RFE-GS, SVM-RFE-PSO, and SVM-RFE-GA algorithm on TCGA data set.

Measure	SVM-RFE-GS	SVM-RFE-PSO	SVM-RFE-GA
genes	15	6	8
Accuracy	91.0017%	91.6808%	91.3413%
Precision	99.2593%	100%	99.6296%
Recall	91.6239%	91.6808%	91.6525%
F-score	95.2889%	95.6599%	95.4747%
AUC	0.79603	0.87487	0.53023

**Table 8:** The performance comparison of RFFS, RFFS-GS, and mRMR algorithm on GEO data set.

Measure	RFFS	RFFS-GS	mRMR
genes	20	18	12
Accuracy	76.9231%	80.0000%	72.3077%
Precision	73.5294%	76.4706%	64.7059%
Recall	80.6452%	83.871%	78.5714%
F-score	76.9231%	80.0000%	70.9677%
AUC	0.75568	0.74242	0.70644

**Table 9:** The performance comparison of RFFS, RFFS-GS, and mRMR algorithm on TCGA data set.

Measure	RFFS	RFFS-GS	mRMR
genes	20	15	12
Accuracy	91.6808%	92.1902%	91.8506%
Precision	100%	97.9630%	100%
Recall	91.6808%	93.7943%	91.8367%
F-score	95.6599%	95.8333%	95.7447%
AUC	0.61494	0.78893	0.85408

**Table 10:** The classification accuracy of other algorithms on GEO data set.

Measure	Accuracy
SVM-RFE-CV	73.85%
LS-SVM	68.42%
PCA+FDA	66.92%
MI+SVM	62.57%
SVM-RFE-GS	78.46%
SVM-RFE-PSO	81.54%
SVM-RFE-GA	76.92%

**Table 11:** The information of eight genes screened by the SVM-RFE-PSO algorithm on GEO data set.

Probe ID	Gene symbol	Gene ID
100694	SLC27A3	hCG40629.3
175122	TUBA1B	hCG2036947
149826	METTL3	hCG2014575
203507	TTYH3	hCG18437.3
158666	CHST14	hCG1647400.1
147893	REPS1	hCG18282.3
104157	PEMT	hCG31440.2
119326	ANXA7	hCG18031.2

**Table 12:** The information of six genes screened by the SVM-RFE-PSO algorithm on TCGA data set.

Gene symbol	Gene ID
ABO	28
ACAT2	39
ACCN3	9311
ACCN1	40
ABCD3	5825
ACADSB	36

**Table 13:** The information of screened genes on two data sets.

Gene symbol	GEO data set	TCGA data set
Gene 1	SLC27A3	ABO
Gene 2	TUBA1B	ACAT2
Gene 3	METTL3	ACCN3
Gene 4	TTYH3	ACCN1
Gene 5	CHST14	ABCD3
Gene 6	REPS1	ACADSB
Gene 7	PEMT	−
Gene 8	ANXA7	−

## Data Availability

The data used to support the findings of this study are available from the corresponding author upon request.

## Supplementary Materials

Supplementary Materials The supplementary materials contain the files of GEO data set and TCGA data set used in the experiment, as well as the main code files used in the experiment.
